# The influence of red pepper powder on the density of *Weissella koreensis* during kimchi fermentation

**DOI:** 10.1038/srep15445

**Published:** 2015-10-26

**Authors:** Bo Kyoung Kang, Min Seok Cho, Tae-Young Ahn, Eui Seok Lee, Dong Suk Park

**Affiliations:** 1 Department of Agricultural Biotechnology, National Academy of Agricultural Science, Rural Development Administration, Jeonju 560-500, Republic of Korea; 2 Department of Microbiology, Dankook University, Cheonan 330-714, Republic of Korea; 3 Department of Oral and Maxillofacial Surgery, Guro Hospital, Korea University, Seoul 152-703, Republic of Korea

## Abstract

*Weissella koreensis* is a psychrophilic bacterium that is the dominant species found in kimchi and exhibits anti-obesity effects via its production of ornithine. In this study, we mined the genome of *W. koreensis* KACC15510 to identify species-specific genes that can serve as new targets for the detection and quantification of *W. koreensis* in kimchi. A specific polymerase chain reaction (PCR) primer set for the membrane protein-encoding gene of *W. koreensis* KACC15510 was designed and investigated to quantify its sensitivity and specificity for detecting the bacterium in kimchi. The specificity of the primer set was evaluated using genomic DNA from eight isolates of *W. koreensis*, 11 different species of *Weissella* and 13 other reference lactic acid bacterium (LAB) strains. In addition, red pepper powder was observed to strongly influence the density of *W. koreensis* during kimchi fermentation.

*Weissella koreensis* is a Gram-positive, non-spore-forming, heterofermentative, and nonmotile short-rod bacterium that belongs to the family Leuconostocaceae^1^. *W. koreensis* is the predominant lactic acid bacterium (LAB) isolated from kimchi, a traditional Korean food composed of fermented vegetables^2^. As a psychrophilic bacterium, *W. koreensis* produces D-(-)-lactic acid and metabolites from glucose, which contribute to kimchi’s taste and flavor^3^ and to sourdough fermentation during bread-making. An earlier study has also reported that *W. koreensis* inhibits the germination of target microorganism spores during food fermentation and exhibits an anti-obesity effect by producing the non-protein amino acid (a.a.) ornithine^3^. Kimchi is a traditional fermented vegetable food emblematic of Korean culture that is fermented from vegetables such as Chinese cabbage and radish. Currently, kimchi is industrially produced via fermentation and is now consumed as a side dish worldwide. The most common type of whole kimchi (baechu-kimchi) is made by mixing salted white cabbage with a kimchi paste made of red pepper powder (*Capsicum annuum*), garlic, spring onion, Korean radish ginger, fish sauce (salted seafood), starch paste (made of rice or wheat starch) and other ingredients, such as fresh seafood. White kimchi (baek-kimchi made from Chinese cabbage) and watery kimchi (mul-kimchi, which is made from Chinese cabbage and radish, and dongchimi, which is made from radish) are made without the use of a red pepper powder. These types of kimchi are characterized by many fresh flavours and are extremely refreshing^4^. To date, more than 100 species of LAB and several yeast strains have been identified in kimchi, including *Lactobacillus*, *Leuconostoc*, and *Weissella* species^5^. In particular, *W. koreensis*, a *Weissella* species, has been reported to be the most important microorganism in kimchi and has been used effectively in making whole-wheat bread, together with baker’s yeast^3^.

Thus, establishing an accurate, rapid, sensitive, and practical method based on quantitative polymerase chain reaction (qPCR) to detect and quantify of *W. koreensis* in various fermented foods is necessary. Recently, species, subspecies, and strain-specific deoxyribonucleic acid (DNA) probes have been used extensively to screen, detect, quantify, and identify strains of bacteria, yeast, and viruses^6^. Many molecular assays based on 16S ribosomal RNA (rRNA) and a well-characterized gene that encodes a function relevant for a specific microorganism’s metabolism have been used to detect and identify *Weissella* species, but serious problems with the identification and detection of *W. koreensis* isolates have been identified: these assays also detect other *Weissella* species or do not produce amplicons from *W. koreensis* strains^7^. In addition, many multiplex PCR and chromogenic DNA macroarray systems for simultaneous amplification of several genes in a single assay have been developed. Nevertheless, these methods exhibit limitations: detecting target cells in mixtures with significantly different bacteria ratios or in food samples remains a challenge^7^. Consequently, the detection specificity, which depends on both the uniqueness of the sequence to a bacterium of interest and the specific binding of the primers and probe to the target sequence, is crucial for the efficacy of any PCR detection method.

Over the past 10 years, many efforts have been made to sequence numerous strains of LABs. The increasing number of available LAB genome sequences in databases, together with various bioinformatics tools, provides a resource for the development of more reliable, fast and cost-effective methods for bacterial identification in a wide range of samples. In particular, the genomic information for LABs available in public databases makes it possible to distinguish a target LAB from closely related lineages between species groups. However, despite the major advances in LAB bioinformatics over the last few years by the microorganism industry, methods for detecting, identifying, and quantifying specific LABs remain limited. Therefore, in the present study, we exploited the genome sequence information available in public databases (ftp://ftp.ncbi.nlm.nih.gov/genbank/) to develop a real-time PCR assay for accurate detection and identification of *W. koreensis*. A pair of species-specific primers based on a membrane protein from the genomic sequence of *W. koreensis* KACC15510 was designed.

Bacterial membranes have been reported to perform diverse functions dependent on whether the membrane is specialized or cytoplasmic; the latter exhibit transport, mitochondrial activities and biosynthetic functions that are crucial for the assembly of membranes, walls and capsules. Membrane fusion proteins are found only in the prokaryotic world and function in conjunction with a variety of transport systems in Gram-positive and Gram-negative bacteria. These proteins are functional subunits of multi-component transporters that perform diverse physiological functions in both Gram-positive and Gram-negative bacteria. Bacterial membrane proteins are diverse in structure and function and vary significantly in size, with residue lengths that range from 200 to 650 a.a.^8^.

In this study, we established a reliable and efficient procedure for quantitative detection of *W. koreensis* in kimchi samples via SYBR Green PCR. Our results revealed that this SYBR Green qPCR-based method can be used for the specific detection and quantification of *W. koreensis* in various products. Using this real-time quantitative PCR assay, we found that red pepper powder greatly influences the density of *W. koreensis* during kimchi fermentation.

## Results

### Specificity of the designed primer set

A species-specific primer set was designed based on sequences of a membrane protein-encoding gene of *W. koreensis* KACC15510 (GenBank accession No. WP_013989464.1). The specificity of the primer sequences was tested using Basic Local Alignment Search Tool (BLAST) searches and electronic PCR (e-PCR) analysis (http://www.ncbi.nlm.nih.gov/). The BLASTn search exhibited no significant matches with previously reported sequences, with the exception of genomic DNA from *W. koreensis* KACC15510. The BLASTx searches for the predicted protein sequence revealed that *W. confusa* (GenBank accession No. WP_003607668.1) exhibited the closest similarity to the membrane protein. However, the PCR-amplified DNA sequence exhibited no significant matches in either the Nucleotide BLAST (BLASTn) or Align Sequences Translated BLAST (BLASTx) searches.

**Figure 1:**
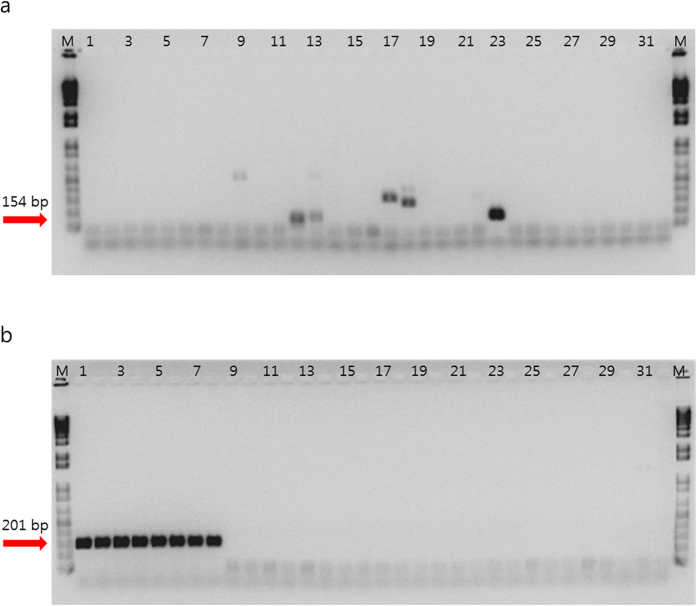
PCR amplification of maltose phosphorylase and the membrane protein. Lane M is the size marker (1 kb DNA plus ladder; Gibco BRL), lanes 1 to 8 are *W. koreensis* strains, lanes 9 to 31 are included strains from other *Weissella* species along with strains from species of *Lactobacillus* and *Leuconostoc*, as specified in Table 1, and lane 32 is a negative control (distilled water). (**a**) Maltose phosphorylase gene^7^. (**b**) The membrane protein-encoding gene used in this study.

The species-specific molecular marker, a membrane protein-encoding gene, was amplified using the WK201F/R primers. Their specificity was validated against 11 different species of *Weissella* and 13 other reference LAB strains. All 8 *W. koreensis* strains consistently tested positive, regardless of the presence of other species of the bacterium in the sample, and only *W. koreensis* strains produced a single amplified product of 201 bp. In contrast, a PCR assay that targeted maltose phosphorylase did not distinguish *W. koreensis* from the other tested bacterial species listed in Table 1 (see Fig. 1).

### Standard curves and melting temperature

**Figure 2:**
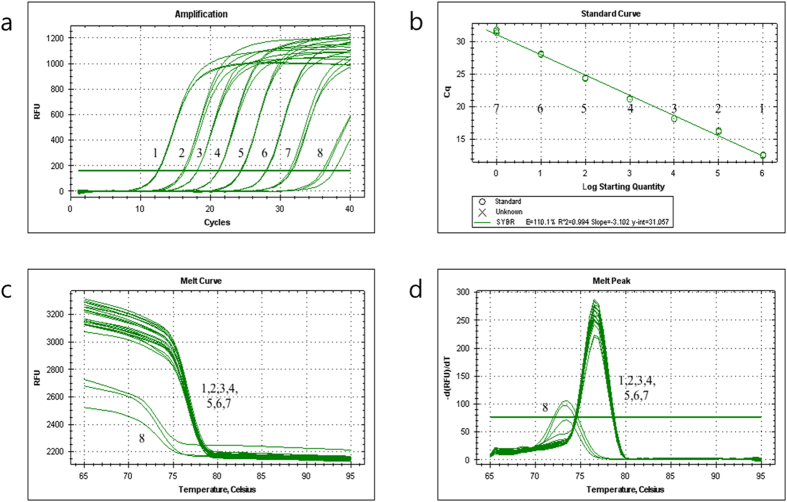
Specificity, melting peak and standard curve of the WK201F/R primer set with SYBR Green qPCR. (**a**) The fluorescence intensity as a function of the concentration of template. For each assay, a series of 10-fold dilutions of cloned DNA (range of 1.42 × 10^3^ to 1.42 × 10^9^ copies/μl) was used as the template for PCR (1–7, sample dilutions; 8, no-template control). (**b**) Standard curve derived from the amplification plot. (**c**) Melting curve analysis (1–7, sample dilutions; 8, no-template control). (**d**) Melting peak analysis (1–7, sample dilutions; 8, no-template control). The amplified products’ derivative of relative fluorescence units [-d(RFU)/dT] is plotted as a function of temperature (amplified product, 77.0 °C). The high peak indicates the amplified product, and the low peak is the no-template control.

We used SYBR Green real-time PCR analysis of *W. koreensis* to generate a standard curve by plotting the mean cycle threshold (Ct; n = 3) versus the logarithmic concentration of genomic DNA, cloned DNA, and the density of the cell suspension (ranges of 5 × 10^0^ to 5 × 10^–6^ ng/μl, 1.42 × 10^9^ to 1.42 × 10^3^ copies/μl, and 1.28 × 10^9^ to 1.28 × 10^4^ CFU/ml, respectively; Fig. 2a and Table 2). The limit of quantitation (LOQ) assay exhibited a good linear response and high correlation coefficient (R^2^ = 0.994). A standard curve analysis of the linear portion of the slope resulted in a coefficient of −3.102, which yielded a PCR efficiency of 110.1% and a y-intercept value of 31.057 (Fig. 2b). The melting curve derived from the amplification plot is shown in Fig. 2c, and the analysis of the melting temperature and melting peaks of *W. koreensis* using SYBR Green qPCR revealed a reproducible melting temperature of 77.0 °C and specific peaks (Fig. 2d). A standard curve of genomic DNA and the bacterial cell suspension exhibited a linear correlation between the Ct values and the concentrations of input DNA (R^2^ = 0.999, slope = −3.302) and the bacterial cell suspension (R^2^ = 0.996, slope = −3.414). In addition, an analysis of genomic DNA and the bacterial cell suspension indicated that the detection limit of SYBR Green qPCR was 5 fg/μl (fg per μl reaction mix), which corresponds to 1.28 × 10^4^ CFU/ml (CFU per ml reaction mix) of *W. koreensis* (Table 2).

### Variation in the density of *Weissella koreensis* during kimchi fermentation

**Figure 3:**
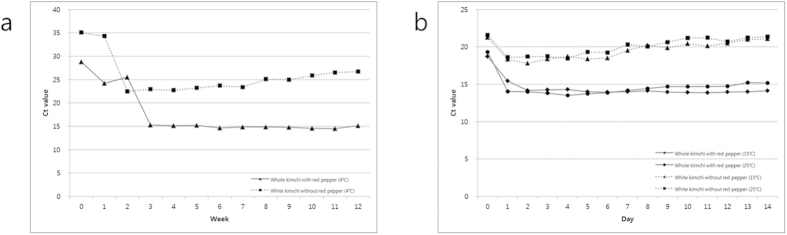
Changes in the Ct value of real-time PCR for the quantification of *W. koreensis* in total DNA from two types of salted Chinese cabbage kimchi fermented at 4 °C (**a**), 15 °C and 25 °C (**b**).

Two types of salted Chinese cabbage kimchi - whole kimchi with red pepper powder and white kimchi without red pepper powder - were obtained from a kimchi company in Korea, and samples of each were stored at 4, 15 and 25 °C. The density of *W. koreensis* in the kimchi samples preserved at 4, 15, and 25 °C varied. The samples taken from whole kimchi stored at 4 °C exhibited the lowest Ct value between weeks 1 and 3 compared with those from white kimchi under the same conditions (Fig. 3a). However, whole kimchi stored at 15 or 25 °C presented the lowest Ct value between days 1 and 2 (Fig. 3b). Thus, red pepper powder was observed to strongly influence the density of *W. koreensis* during kimchi fermentation, regardless of the temperature and fermentation period (Fig. 3).

## Discussion

Probiotic bacteria have been historically considered to hold great promise for the treatment of gastrointestinal disorders. However, further studies are required to create a more scientific basis for the action of probiotics. Although unprecedented levels of scientific evidence supporting the health benefits of LABs and their products have been accumulated, no clear evidence describing the role of a specific bacterium has been presented. Hence, establishing reliable and efficient species-specific molecular probes for quantitative detection of targeted LABs used in various lactic acid products is critical because such probes would enable detection of individual species and overall profiling of changes in the community structure in response to changes in variables such as time and temperature.

In the initial studies of kimchi LAB communities, traditional approaches based on morphological and phenotypic identification of LAB species grown on agar media were used, but such studies were often unsuccessful^9^. This method has major disadvantages, such as the long assay time of 10 days and the possibility of detecting cultivable cells only. For these reasons, molecular methods that use sequences of 16S ribosomal RNA genes and other genes for the identification of isolated strains have attracted the attention of many researchers^10^. However, these approaches are not suitable for monitoring the succession of targeted LAB during kimchi fermentation^10^. Thus, the value of molecular detection and quantification methods for studying LAB is immense.

Fortunately, the number of microbial genome sequences available has increased dramatically. In particular, the availability of complete or draft LAB genome sequences presents a great opportunity for improving existing molecular detection and quantification tools by identifying new targets for more sensitive and specific detection.

Recently, among the LABs found in kimchi - including *L. plantarum*, *L. sake*, *Leu. mesenteroides*, *Leu. lactis*, *Leu. citreum*, *Pediococcus pentosaceus*, *W. cibaria*, and *W. confusa* - *W. koreensis* has been reported to be associated with L-Ornithine production from arginine and to play a crucial role in preventing intracellular lipid accumulation by down-regulating the expression of adipocyte-specific genes. L-Ornithine is a medicinal, non-protein a.a. that has the potential to combat obesity by promoting hormone release and accelerating the rate of basal metabolism^2^.

In addition, of the many bioactive materials found in kimchi, capsaicin derived from red pepper powder has been proposed as an effective agent for fat digestion. However, little information regarding the correlation between red pepper powder and *W. koreensis* exists. Therefore, we explored species-specific genes using BLAST searches and developed a primer set to investigate this correlation. The species-specific primer set was derived from the whole-genome sequence of *W. koreensis* KACC15510 (GenBank accession no. WP_013989464.1). We focused on a membrane protein-encoding gene that was confirmed to be highly variable among *Weissella* species (Fig. 1).

In a previous report^11^, the proportion of *Weissella* was found to be higher in kimchi with red pepper powder than in kimchi without red pepper powder, whereas the proportions of *Leuconostoc* and *Lactobacillus* were lower in kimchi with red pepper powder. However, red pepper powder had little influence on the cell density of *L. plantarum* in kimchi, whereas temperature greatly impacted the proportion of *L. plantarum* (data not shown). Consequently, red pepper powder is estimated to greatly influence the growth of particular LABs in kimchi. As shown in Fig. 3, in this study, the proportion of *W. koreensis* was much greater in whole kimchi with red pepper powder than in white kimchi without red pepper powder, regardless of temperature and fermentation period. At 4 °C, the differences in proportion of *W. koreensis* were more apparent than at 15 and 25 °C.

*W. koreensis* was verified to be the dominant species and could ferment kimchi at temperatures as low as −1 °C^12^. In addition, *W. koreensis* was confirmed to exhibit relatively good psychrophilic growth, which predominated at 4 °C or colder. However, *Leuconostoc* species incubated at 15 or 25 °C did not delay the growth of *W. koreensis* (data not shown). As shown in Fig. 3, the patterns for the changing populations of *W. koreensis* were similar at 4, 15 and 25 °C. The populations of *W. koreensis* exhibited a rapid increase during the early fermentation period and then remained constant regardless of temperature.

Consequently, to standardize the ripening process during quality-controlled kimchi production, accurate monitoring of changes in the microbial community *in situ* during the fermentation period is essential^13^.

The key advantages of this newly developed assay are its specificity and rapidity: it allows species-specific identification and quantification of *W. koreensis* strains within 1 h without any prior cultivation. The number of replicates used to calculate standard curves and the small standard error among these replicates ensure that the assay is reproducible and highly robust, even with DNA isolated from kimchi.

In conclusion, this newly developed real-time PCR assay detects *W. koreensis* with high specificity and sensitivity and without false-positive signals from other LABs in pure cultures and in DNA mixtures extracted from kimchi. This real-time PCR assay may be a useful method for detection and quantification of *W. koreensis* in food for quality management purposes.

## Methods

### Bacterial strains, growth conditions, and DNA preparation

The bacterial strains were obtained from the Korean Agricultural Culture Collection (KACC) in the Republic of Korea and the Belgian Co-ordinated Collections of Micro-organisms (BCCM^TM^) (Table 1). All strains were grown on de Man Rogosa Sharpe (MRS) agar (Oxoid, Basingstoke, Hampshire, U.K.) plates at 25 to 30 °C for 48 h. Total genomic DNA was extracted using a bacterial genomic DNA extraction kit from Qiagen (Hilden, Germany). To measure the quantity and purity of genomic DNA, a NanoDrop ND-1000 spectrophotometer (NanoDrop Technologies, Wilmington, DE, USA) was used.

### Kimchi sample preparation for PCR

To test the quantitative analysis of kimchi samples, 20 ml of kimchi soup was periodically taken from the two types of kimchi. These were also used for the conventional or real-time PCR analyses. Each kimchi soup sample was filtered through four layers of sterile coarse gauze (Daehan, Daejeon, Korea) to remove large slices and was spun down at 13,000 rpm at 4 °C for 10 min. DNA was extracted using a Fast DNA^®^ spin kit for soil (MP Biomedicals, Solon, OH, USA).

### Genome analysis and primer design

The genome sequences of *W. koreensis* KACC15510 and the other LABs used in this study were downloaded from the NCBI bacterial genome database (ftp://ftp.ncbi.nlm.nih.gov/genomes/bacteria/) and compared using a modified computational pipeline^14^,^15^. On the basis of these comparative outputs, target genes that had no significant similarity in nucleotide sequence among other LABs were selected as PCR targets. A species-specific primer set based on a membrane protein-encoding gene of *W. koreensis* KACC15510 (GenBank accession No. WP_013989464.1) with a predicted PCR product of 201 bp was designed. Amplification primers for conventional PCR and SYBR Green qPCR were synthesized by Bioneer Corporation (Daejeon, Korea) (Table 3).

### Conventional PCR

The PCR amplifications were performed with the above primers (0.2 μM final concentration) and GoTaq^®^ Flexi DNA polymerase (1 × buffer, 4.0 mM MgCl_2_, 0.2 mM of each dNTP, GoTaq^®^ DNA polymerase 1.25 U final concentration; Promega, Madison, WI, USA) in a final volume of 25 μl according to the manufacturer’s instructions; 25 ng of genomic DNA from a given bacterial strain were used. Amplifications were performed in a PTC-225 thermocycler (MJ Research, Watertown, MA, USA) with the following cycling conditions: initial denaturation of 5 min at 95 °C; 35 cycles of 1 min at 95 °C, 30 s at 61 °C, and 1 min at 72 °C; and a final cycle extension of 7 min at 72 °C. After the PCR reaction, each amplified PCR product was electrophoresed through a 1.5% (wt/vol) agarose gel stained with ethidium bromide (EtBr), was visualized on an ultraviolet (UV) transilluminator and was imaged using a VersaDoc 1000 gel imaging system (Bio-Rad laboratories, Hercules, CA, USA).

### SYBR Green qPCR

The SYBR Green qPCR assay was performed in a 20 μl reaction. All amplifications were performed with the WK201F/R primers (0.5 μM final concentration), iQ^TM^ SYBR^®^ Green Supermix (Bio-Rad Laboratories, Hercules, CA, USA), and approximately 5 ng of purified DNA from each sample. Real-time PCR amplifications were performed using a CFX96 real-time PCR system (Bio-Rad Laboratories, Hercules, CA, USA) with the following cycling conditions: initial denaturation of 30 s at 95 °C; 40 cycles of 5 s at 95 °C and 30 s at 61 °C; and a melting curve analysis from 65 to 95 °C with an increment of 0.5 °C per 5 s. The determination of the Ct and the data analysis were performed automatically using the CFX Manager^TM^ Version 1.6 software package (Bio-Rad Laboratories, Hercules, CA, USA).

The LOQ and limit of detection (LOD) of the SYBR Green qPCR assay were determined using 10-fold dilutions of plasmid DNA, genomic DNA and a bacterial cell suspension of *W. koreensis* KACC11853 in a 20 μl reaction mixture containing 10 μl of iQ^TM^ SYBR^®^ Green Supermix (Bio-Rad Laboratories, Hercules, CA, USA) and 5 pM each of WK201F/R primers. This LOQ and LOD were reproducible with serial dilutions and SYBR Green qPCR testing in triplicate. The copy number of the plasmid DNA was calculated using the following equation^16^:


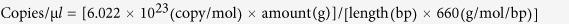


## Additional Information

**How to cite this article**: Kang, B. K. *et al*. The influence of red pepper powder on the density of *Weissella koreensis* during kimchi fermentation. *Sci. Rep*. **5**, 15445; doi: 10.1038/srep15445 (2015).

## Figures and Tables

**Table 1:** Bacterial strains used in the PCR specificity test.

No.	Bacterial strains	Source	Origin	This study
1	*Weissella koreensis*	KACC 11853^T^	Kimchi	+^a^
2	*Weissella koreensis*	KACC 17102	Cabbage Kimchi	+
3	*Weissella koreensis*	KACC 17103	Cabbage Kimchi	+
4	*Weissella koreensis*	KACC 17104	Radish Kimchi	+
5	*Weissella koreensis*	KACC 17870	Kimchi	+
6	*Weissella koreensis*	KACC 17872	Kimchi	+
7	*Weissella koreensis*	KACC 17873	Kimchi	+
8	*Weissella koreensis*	KACC 17874	Kimchi	+
9	*Weissella kandleri*	LMG 18979^T^	Desert spring	−
10	*Weissella viridescens*	KACC 11850^T^	Cured meat products	−
11	*Weissella minor*	KACC 13437^T^	Slime from milking machine	−
12	*Weissella soli*	KACC 11848^T^	Garden soil	−
13	*Weissella halotolerans*	KACC 11843^T^	Sausage	−
14	*Weissella cibaria*	KACC 11862^T^	Chili bo	−
15	*Weissella confusa*	KACC 11841^T^	Saccharum officinarum	−
16	*Weissella hellenica*	KACC 11842^T^	Naturally fermented Greek sausage	−
17	*Weissella thailandensis*	KACC 11849^T^	Fermented fish	−
18	*Weissella paramesenteroides*	KACC 11847^T^	N.D.	−
19	*Lactobacillus brevis*	KACC 11433^T^	Human, faeces	−
20	*Lactobacillus curvatus*	KACC 12415^T^	Milk	−
21	*Lactobacillus kimchii*	KACC 12383^T^	Kimchi	−
22	*Lactobacillus paraplantarum*	KACC 12373^T^	Beer	−
23	*Lactobacillus pentosus*	LMG 10755^T^	N.D.	−
24	*Lactobacillus plantarum*	LMG 6907^T^	Pickled cabbage	−
25	*Lactobacillus sakei*	KACC 12414^T^	Starter of sake (Moto)	−
26	*Leuconostoc carnosum*	KACC 12255^T^	Vacuum-packed beef stored at low temperature	−
27	*Leuconostoc citreum*	KACC 11860^T^	Honeydew of rye ear	−
28	*Leuconostoc gelidum*	KACC 12256^T^	Vacuum-packed meat	−
29	*Leuconostoc inhae*	KACC 12281^T^	Kimchi	−
30	*Leuconostoc lactis*	KACC 12305^T^	Milk	−
31	*Leuconostoc mesenteroides*	KACC 12312^T^	Fermenting olives	−

KACC, Korean Agricultural Culture Collection, Republic of Korea; LMG, The Belgian Co-ordinated Collections of Microorganisms (BCCM^TM^), Belgium.

N.D., not determined.

^T^Type of strain.

^a^+, detected; −, not detected.

**Table 2:** Mean Ct end-point fluorescence of 10-fold serial dilutions of *Weissella koreensis* KACC11853 cloned DNA, genomic DNA and a cell suspension determined with a real-time PCR assay.

Cloned DNA		Genomic DNA		Cell suspension	
Weight/μl reaction mix	Ct ± SD (*n* = 3)	Weight/μl reaction mix	Ct ± SD (*n* = 3)	CFU/ml reaction mix	Ct ± SD (*n* = 3)
5 ng (1.42 × 10^9^ copies)	12.59 ± 0.06	5 ng	14.57 ± 0.10	N.D.^a^	N.D.
500 pg (1.42 × 10^8^ copies)	16.23 ± 0.13	500 pg	17.82 ± 0.05	1.28 × 10^9^	18.23 ± 0.13
50 pg (1.42 × 10^7^ copies)	18.18 ± 0.02	50 pg	21.30 ± 0.16	1.28 × 10^8^	21.21 ± 0.12
5 pg (1.42 × 10^6^ copies)	21.19 ± 0.03	5 pg	24.52 ± 0.18	1.28 × 10^7^	24.81 ± 0.24
500 fg (1.42 × 10^5^ copies)	24.42 ± 0.06	500 fg	27.97 ± 0.11	1.28 × 10^6^	28.14 ± 0.30
50 fg (1.42 × 10^4^ copies)	28.08 ± 0.08	50 fg	31.26 ± 0.29	1.28 × 10^5^	32.15 ± 0.56
5 fg (1.42 × 10^3^ copies)	31.26 ± 0.23	5 fg	34.22 ± 0.68	1.28 × 10^4^	34.90 ± 0.43

^a^N.D., Not determined.

**Table 3:** PCR primers and their target and annealing temperatures of the *W. koreensis* PCR screenings used in this study.

Primer	Oligonucleotide sequence (5′-3′)	Annealing (°C)	Amplicon (bp)	Target gene	Reference
WkoF	GCCGAATTAAGTAGTGTAAAGTCAAATG	60	154	Maltose phosphorylase gene	7
WkoR	TCTGCCGAAGCTTGACCGG				
Wk201F	AAGGTCGCCATTTATTTTTG	61	201	Membrane protein gene	This study
Wk201R	CGTTTATTCCGATCTTTATGG				
